# The RNA-binding protein HuR stabilizes survivin mRNA in human oesophageal epithelial cells

**DOI:** 10.1042/BJ20110028_EOC

**Published:** 2026-07-24

**Authors:** James M. Donahue, Elizabeth T. Chang, Lan Xiao, Peng-Yuan Wang, Jaladanki N. Rao, Douglas J. Turner, Jian-Ying Wang, Richard J. Battafarano

**Affiliations:** 1 Cell Biology Group, Department of Surgery, University of Maryland School of Medicine, Baltimore, MD 21201, U.S.A.; 2 Baltimore Veterans Affairs Medical Center, Baltimore, MD 21201, U.S.A.

**Keywords:** 3′-untranslated region (3′-UTR), Hu antigen R (HuR), mRNA stability, p53, ribonucleoprotein, survivin

The *Biochemical Journal* has been made aware of potential issues surrounding the scientific validity of this paper and is hence issuing a permanent Expression of Concern to notify readers.

A similarity was identified between Figures 2A and 3A, whereby both figure panels have the same HuR bands but different associated loading controls. We have been unable to get in touch with the corresponding author or first author with regard to this similarity, and whilst we received a response from one of the co-authors of the article, they were unable to provide any raw data or an explanation.

The Editorial Board has therefore decided to issue a permanent Expression of Concern for this article to alert readers to our concerns.

